# Editorial: Nutrition and muscle recovery after exercise

**DOI:** 10.3389/fspor.2024.1413822

**Published:** 2024-04-23

**Authors:** Stephen M. Cornish, Matthew J. Barnes

**Affiliations:** ^1^ Faculty of Kinesiology and Recreation Management, University of Manitoba, Winnipeg, MB, Canada; ^2^ School of Sport, Exercise & Nutrition, Massey University, Palmerston North, New Zealand

**Keywords:** nutrition, recovery, exercise-induced muscle damage (EIMD), inflammatory markers, metabolism

**Editorial on the Research Topic** Nutrition and muscle recovery after exercise

Nutrition plays a critical role in the recovery of skeletal muscle after exercise. Adequate macronutrient intake, particularly protein, is essential to support acute muscle repair, growth and eventual adaptation, while metabolic recovery depends on energy substrate availability. Additionally, while certain micronutrients may aid in skeletal muscle growth and repair, the evidence for this is less prevalent in the scientific literature. Skeletal muscle's ability to recover from exercise may also be influenced by nutrient timing, type, and quantity. Therefore, research in this area is crucial to improve our understanding of the role of nutrition in recovery of skeletal muscle after exercise and to identify optimal nutritional strategies to enhance exercise performance, recovery and adaptation.

This research topic aimed to cover a wide range of topics related to the interaction between nutrition and exercise, including recovery from exercise-induced muscle damage (EIMD), the impact of different nutrients on muscle recovery, nutrient timing, dietary interventions to enhance recovery, and the role of nutrition in optimizing exercise performance and adaptation.

Four studies are included in this research topic; three original studies investigating the influence of various protein sources on recovery from EIMD, and a review outlining the potential benefits of hydrogen enriched water for altering post-exercise oxidative stress.

Although there is considerable research supporting the use of cow's milk for recovery post-eccentric exercise, the potential of sheep's milk, which is more nutrient rich and potentially easier on the gut, had not previously been investigated. As such, Ravenwood et al. used a double blind, randomized, cross-over design to compare the effects of consuming 500 ml of chocolate flavored sheep's and cow's milk on muscle function and soreness in the days after strenuous eccentric exercise. Despite providing more energy and protein, recovery with sheep's milk was no better, or worse, than the effects of cow's milk suggesting the two milks may be equally beneficial for recovery. Sheep's milk was more satiating than cow's milk, however neither milk negatively impacted measures of gut discomfort.

While many studies have investigated the acute benefits of nutritional interventions on recovery, the regular consumption of food or supplements alone or in combination with exercise may enhance adaptation to exercise and provide protection against EIMD, when it does occur. In a randomized, cross over study, Siegel et al. investigated whether consuming 57 g of whole almonds daily for eight weeks could alleviate symptoms of EIMD, and improve cardiometabolic health, mood and appetite, compared to a control. Although, post-exercise changes in creatine kinase concentration and isokinetic force were not different between treatments, consuming almonds reduced EIMD muscle soreness and maintained performance during the vertical jump, suggesting that almonds may help maintain or improve exercise tolerance and performance in middle-aged adults.

Bischof et al. investigated whether supplementing 12 weeks of concurrent training with 15 g of specific collagen peptides could alter responses to EIMD, compared to a placebo. Participants were separated into two groups (specific collagen peptides or placebo) and completed a bout of 150 drop jumps before and after the training period. The combination of specific collagen peptides and concurrent training reduced the impact of EIMD on muscle function and improved the rate of recovery after the second bout of drop jumps, in particular voluntary force production, rate of force development and counter movement jump height. These findings suggest that regularly consuming specific collagen peptides, alongside exercise, can enhance muscular adaptations that lead to a greater regenerative capacity in skeletal muscle.

Strenuous exercise brings about oxidative stress that may overwhelm the endogenous antioxidant system leading to fatigue, inflammation and cellular damage. Therefore, nutritional strategies to combat oxidative stress may prove beneficial to recovery and subsequent exercise performance. The novel use of hydrogen enriched water to alter the post-exercise oxidative state was reviewed by Li et al. There is limited research on this novel approach, with six studies included in the review. However, the reviewed studies suggest that, while hydrogen enriched water does not directly inhibit oxidative stress, it may enhance antioxidant potential, particularly after high intensity interval training. Given its potential, more research on the use of hydrogen enriched water for recovery appears to be warranted.

The articles included in this research topic reflect the potential of a variety of foods, taken acutely or habitually, to benefit muscle recovery after exercise.

